# Estimated glomerular filtration rate decline and risk of end-stage renal disease in type 2 diabetes

**DOI:** 10.1371/journal.pone.0201535

**Published:** 2018-08-02

**Authors:** Megumi Oshima, Tadashi Toyama, Masakazu Haneda, Kengo Furuichi, Tetsuya Babazono, Hiroki Yokoyama, Kunitoshi Iseki, Shinichi Araki, Toshiharu Ninomiya, Shigeko Hara, Yoshiki Suzuki, Masayuki Iwano, Eiji Kusano, Tatsumi Moriya, Hiroaki Satoh, Hiroyuki Nakamura, Miho Shimizu, Akinori Hara, Hirofumi Makino, Takashi Wada, The Research Group of Diabetic Nephropathy, Ministry of Health, Labour, and Welfare of Japan

**Affiliations:** 1 Department of Nephrology, Kanazawa University Hospital, Kanazawa, Japan / Department of Disease Control and Homeostasis, Institute of Medical, Pharmaceutical and Health Sciences, Kanazawa University, Kanazawa, Japan; 2 Department of Medicine, Asahikawa Medical University, Asahikawa, Japan; 3 Division of Nephrology and Hypertension, Diabetes Center, Tokyo Women’s Medical University School of Medicine, Tokyo, Japan; 4 Jiyugaoka Medical Clinic, Internal Medicine, Obihiro, Japan; 5 Dialysis Unit, University Hospital of the Ryukyus, Nishihara, Okinawa, Japan; 6 Department of Medicine, Shiga University of Medical Science, Otsu, Shiga, Japan; 7 Department of Medicine and Clinical Science, Graduate School of Medical Sciences, Kyushu University, Fukuoka, Japan; 8 Center of Health Management, Toranomon Hospital, Tokyo, Japan; 9 Health Administration Center, Niigata University, Niigata, Japan; 10 Department of Nephrology, Faculty of Medical Sciences, University of Fukui, Fukui, Japan; 11 Division of Nephrology, Department of Internal Medicine, Jichi Medical University, Tochigi, Japan; 12 Health Care Center, Kitasato University, Sagamihara, Japan; 13 Department of Metabolism and Endocrinology, Juntendo University Graduate School of Medicine, Tokyo, Japan; 14 Department of Environmental and Preventive Medicine, Institute of Medical, Pharmaceutical and Health Sciences, Kanazawa University, Kanazawa, Japan; 15 Department of Medicine and Clinical Science, Okayama University Graduate School of Medicine, Dentistry and Pharmaceutical Sciences, Okayama, Japan; International University of Health and Welfare, School of Medicine, JAPAN

## Abstract

**Background:**

According to studies by the National Kidney Foundation and Food and Drug Administration, 30% and 40% declines in estimated glomerular filtration rate (eGFR) could be used as surrogate endpoints of end-stage renal disease (ESRD). However, the benefits of using these endpoints in diabetic patients remain unclear.

**Methods:**

This cohort study comprised Japanese patients with type 2 diabetes; those with repeated serum creatinine measurements during a baseline period of 2 years (n = 1868) or 3 years (n = 2001) were enrolled. Subsequent risks of ESRD following eGFR declines were assessed.

**Results:**

In the 2-year baseline analysis, the cumulative prevalence of −20%, −30%, −40%, and −53% changes in eGFR were 23.9%, 11.1%, 6.8%, and 3.7%, respectively. There were 133 cases (7.1%) of subsequent ESRD during a median follow-up period of 6.5 years. In the 3-year baseline analysis, the corresponding proportions were 28.1%, 14.0%, 7.7%, and 3.9%, respectively, with 110 participants (5.5%) reaching ESRD during a median follow-up period of 5.5 years. The adjusted hazard ratios of subsequent ESRD following −53%, −40%, −30%, and −20% changes in eGFR during the 2-year baseline period were 22.9 (11.1–47.3), 12.8 (6.9–23.7), 8.2 (4.3–15.5), and 3.9 (2.2–7.0), respectively when compared with the no changes in eGFR. In the 3-year baseline analysis, the corresponding risks were 29.7 (10.8–81.9), 18.4 (7.6–44.7), 12.8 (5.2–32.2), and 5.4 (2.3–12.8), respectively. In the subgroup analysis, similar trends were observed in patients with macroalbuminuria at baseline.

**Conclusions:**

Declines in eGFR were strongly associated with subsequent risk of ESRD in Japanese type 2 diabetic patients. In addition to 30% and 40% declines, a 20% decline in eGFR over 2 years could be considered as a candidate surrogate endpoint of ESRD in diabetic kidney disease.

## Introduction

Diabetes mellitus is the leading cause of chronic kidney disease (CKD) worldwide [1]. In fact, approximately 40% of patients with diabetes develop diabetic kidney disease (DKD) resulting in albuminuria, reduction of estimated glomerular filtration rate (eGFR), or both [2]. DKD is associated with increased risks of end-stage renal disease (ESRD) and cardiovascular mortality independent of other cardiovascular risk factor [3–6]. Hence, in order to establish the best practices for DKD, clinical studies requiring less time and costs need to be conducted.

Several clinical trials using established endpoints such as kidney failure and doubling of serum creatinine (sCr) level, which corresponds approximately to a 57% decline in eGFR using the 2009 CKD-EPI (CKD Epidemiology Collaboration) creatinine equation, have been conducted [7,8]. However, these are late-stage events in CKD requiring large sample sizes and long durations of follow-up for adequate statistical power [9–11].

Recently, the National Kidney Foundation (NKF) and Food and Drug Administration (FDA) committee has published a series of studies to determine whether lesser eGFR declines can be used as a substitute for the doubling of sCr levels among composite kidney endpoints [7, 12–14]. The committee concluded that a 30% decline in eGFR could be considered as an acceptable surrogate endpoint in some circumstances, although a 40% eGFR decline would demonstrate a relatively stronger evidence; they also mentioned that follow-up periods of at least 2 to 3 years were required for both 30% and 40% eGFR declines [10, 15, 16]. These alternative endpoints provide a greater number of events of shorter duration when compared to using the doubling of sCr levels, thus contributing to a reduction in sample size and follow-up period required for the clinical trials.

In Japanese patients with reduced eGFR, 30% and 40% declines over a period of 1 to 2 years were reported to be strongly associated with the risk of ESRD [17]. In the general Japanese population, less than −30% change in eGFR over 2 or 3 years could be considered as a candidate surrogate endpoint for ESRD [18]. Several studies indicated that these associations were consistent across diabetes [7, 12, 16, 17]; however, studies focusing on diabetic patients have not been conducted so far. Therefore, the aim of the current study was to assess the usefulness of 30% and 40% reductions in eGFR over a period of 2 to 3 years as surrogate endpoints for ESRD in DKD, in a Japanese type 2 diabetes cohort.

## Materials and methods

### Study design and population

This cohort study comprised 4814 Japanese patients with type 2 diabetes, who were treated at 10 centers between 1985 and 2010. We diagnosed type 2 diabetes mellitus according to the Japan Diabetes Society (JDS) criteria [19]. Exclusion criteria were any of the following conditions: age <18 years, type 1 or secondary diabetes, renal transplantation, maintenance dialysis, missing value of covariates such as urinary albumin, glycated hemoglobin (HbA1c) or systolic blood pressure (BP), and refusal to provide informed consent. The current study was restricted to those with repeated sCr measurements within a baseline period of 2 or 3 years. Participants who discontinued the follow-up or achieved ESRD within the baseline period were excluded from the study.

### Measurement of study variables

At the beginning of the baseline period, baseline assessment of patients’ characteristics including laboratory measurements (sCr, HbA1c, and urinary albumin-to-creatinine ratio [UACR]), physical findings, sociodemographic information, and medical history were performed at each center.

Blood pressure was measured in the sitting position. sCr was measured by an enzymatic method and eGFR was estimated using the equation proposed by the Japanese Society of Nephrology [20]. Urine albumin and creatinine levels were ascertained using a turbidimetric immunoassay and an enzymatic method on spot urine samples. HbA1c was measured on non-fasting blood samples, standardized by the JDS (normal range 4.3–5.8%), and certified by the US National Glycohemoglobin Standardization Program (NGSP = JDS + 0.4) [19]. A history of cardiovascular disease (CVD) including coronary heart disease, stroke, cerebral hemorrhage, heart failure, and arteriosclerosis obliterans was considered.

### Categories of albuminuria

Based on the classification of CKD [21], the albuminuria category was classified at baseline as normoalbuminuria (<30 mg/gCr), microalbuminuria (≥30 and <300 mg/gCr), and macroalbuminuria (≥300 mg/gCr). Patients examined in this study were categorized and assessed based on the above classifications.

### Study outcomes

The primary outcome of this study was ESRD, which was defined as the initiation of dialysis or kidney transplantation at each clinical center through 2010. These conditions corresponded to the International Classification of Diseases, 11th version (http://www.who.int/classification/icd/en/). Participants were followed until the onset of the first event or the last day of the observation period after each baseline period.

### Surrogate endpoints

In the current study, we focused on percent changes in eGFR during a baseline period of 2 or 3 years as surrogate endpoints of ESRD among type 2 diabetic patients. According to the reports from the NKF-FDA working group, percent change in eGFR was calculated as follows [7, 12]:

$$\frac{\mathrm{last}\;\mathrm{eGFR}\;\mathrm{at}\;\mathrm{baseline}\;\mathrm{period}–\mathrm{first}\;\mathrm{eGFR}}{\mathrm{first}\;\mathrm{eGFR}}\times 100.$$

The surrogate endpoints were defined as −20%, −30%, −40% or −53% changes in eGFR during the baseline period of 2 or 3 years. Doubling of sCr, the established kidney endpoint, corresponds to a −53% change in eGFR in the Japanese creatinine equation for estimated GFR [19], and is approximately equivalent to a −57% change in eGFR according to the CKD-EPI equation [10].

### Statistical analysis

Each observation commenced at the beginning of the baseline period. The baseline characteristics were summarized according to four categories of percent change in eGFR (≤−53%; >−53% to −30%; >−30% to 0%; and >0% [i.e., increase in eGFR]). Data are expressed as mean ± standard deviation (SD).

We estimated the adjusted hazard ratios (HRs) of subsequent ESRD from models with a spline function of percent changes in eGFR and covariates including age, gender, baseline eGFR, baseline UACR, baseline systolic BP, and a history of CVD. Based on previous reports [10, 12, 17], piecewise linear splines for percent changes in eGFR (knots were placed at −53%, −25%, −10%, and 10%) were fit using 0% change as a reference point. Positive predictive value (PPV) was calculated for each surrogate endpoint. Subsequently, we calculated the percent population attributable risk (%PAR), which indicates the ESRD incidence percentage in the total number participants (reached the surrogate endpoints), using the following formula:

$$\%\mathrm{PAR}=\frac{\mathrm{Incidence}\;\mathrm{in}\;\mathrm{the}\;\mathrm{population}‑\mathrm{Incidence}\;\mathrm{in}\;\mathrm{the}\;\mathrm{unexposed}}{\mathrm{Incidence}\;\mathrm{in}\;\mathrm{the}\;\mathrm{population}}\times 100.$$

The positive predictive value (PPV) was then calculated for each surrogate endpoint. P values were two-sided and those <0.05 were considered statistically significant. All analyses were performed using Stata/IC version 15.0 software (Stata Corp, TX, USA).

### Ethics statement

This study was conducted according to the ethical guidelines for epidemiological research designed by the Japanese Ministry of Education, Culture, Sports, Science and Technology and Ministry of Health, Labor, and Welfare (http://www.lifescience.mext.go.jp/files/pdf/n796_01.pdf). The study was approved by the Kanazawa University ethical committee (approval number 2709).

## Results

### Study population and baseline characteristics

Table 1 shows the baseline characteristics of patients enrolled in this study. Within the 2- and 3-year baseline period, 1868 and 2001 participants repeated the sCr measurements, respectively. In the 2-year baseline analysis, the mean age of the participants was 60 years, 57.3% of population were men, mean eGFR was 77 ± 25 (SD) mL/min/1.73 m^2^, and median urinary albumin creatinine ratio (UACR) was 20 (interquartile range [IQR]; 10, 109) mg/gCr. In the 3-year baseline analysis, the mean age was 60 years, 57.9% were men, mean eGFR was 77 ± 24 mL/min/1.73 m^2^, and median UACR was 19 (IQR 9, 79) mg/gCr.

**Table 1:** Baseline characteristics according to urinary albumin levels during the (a) 2-year or (b) 3-year baseline period.

	Normo albuminuria	Micro albuminuria	Macro albuminuria	All participants
**(a) 2-year baseline period (n = 1868)**				
N	1078	495	295	1868
Age (years; mean [SD])	60 (11.3)	62 (11.3)	59 (11.8)	60 (11.4)
Men (n [%])	578 (53.6)	300 (60.6)	192 (65.1)	1070 (57.3)
UACR (mg/g; median [IQR])	11 (7, 17)	72 (46, 135)	1002 (533, 2378)	20 (10, 109)
eGFR (mL/min/1.73 m^2^; mean [SD])	82 (22.5)	76 (24.2)	57 (24.9)	77 (24.9)
Systolic BP (mmHg; mean [SD])	129 (18.2)	137 (19.8)	143 (20.8)	133 (19.7)
Diastolic BP (mmHg; mean [SD])	76 (29.8)	77 (11.4)	78 (13.0)	76 (24.0)
History of CVD (n [%])	101 (9.4)	56 (11.3)	32 (10.9)	189 (10.1)
HbA1c (%; mean [SD])	8.0 (1.8)	8.2 (1.8)	8.1 (1.8)	8.0 (1.8)
**(b) 3-year baseline period (n = 2001)**				
N	1209	525	267	2001
Age (years; mean [SD])	60 (11.2)	61 (11.1)	59 (11.6)	60 (11.2)
Men (n [%])	671 (55.5)	315 (60.0)	173 (64.8)	1159 (57.9)
UACR (mg/g; median [IQR])	11 (7, 17)	70 (46, 127)	830 (497, 2038)	19 (9, 79)
eGFR (mL/min/1.73 m^2^; mean [SD])	82 (22.4)	77 (24.5)	60 (24.6)	77 (24.3)
Systolic BP (mmHg; mean [SD])	128 (17.6)	137 (19.2)	143 (20.7)	133 (19.3)
Diastolic BP (mmHg; mean [SD])	75 (28.4)	76 (11.6)	79 (12.5)	76 (23.3)
History of CVD (n [%])	104 (8.6)	54 (10.3)	26 (9.7)	184 (9.2)
HbA1c (%; mean [SD])	7.8 (1.8)	8.0 (1.7)	8.0 (1.7)	7.9 (1.8)

UACR, urine albumin-to-creatinine ratio; eGFR, estimated glomerular filtration rate; BP, blood pressure; CVD, cardiovascular disease; SD, standard deviation; IQR, interquartile range.

**Fig 1:**
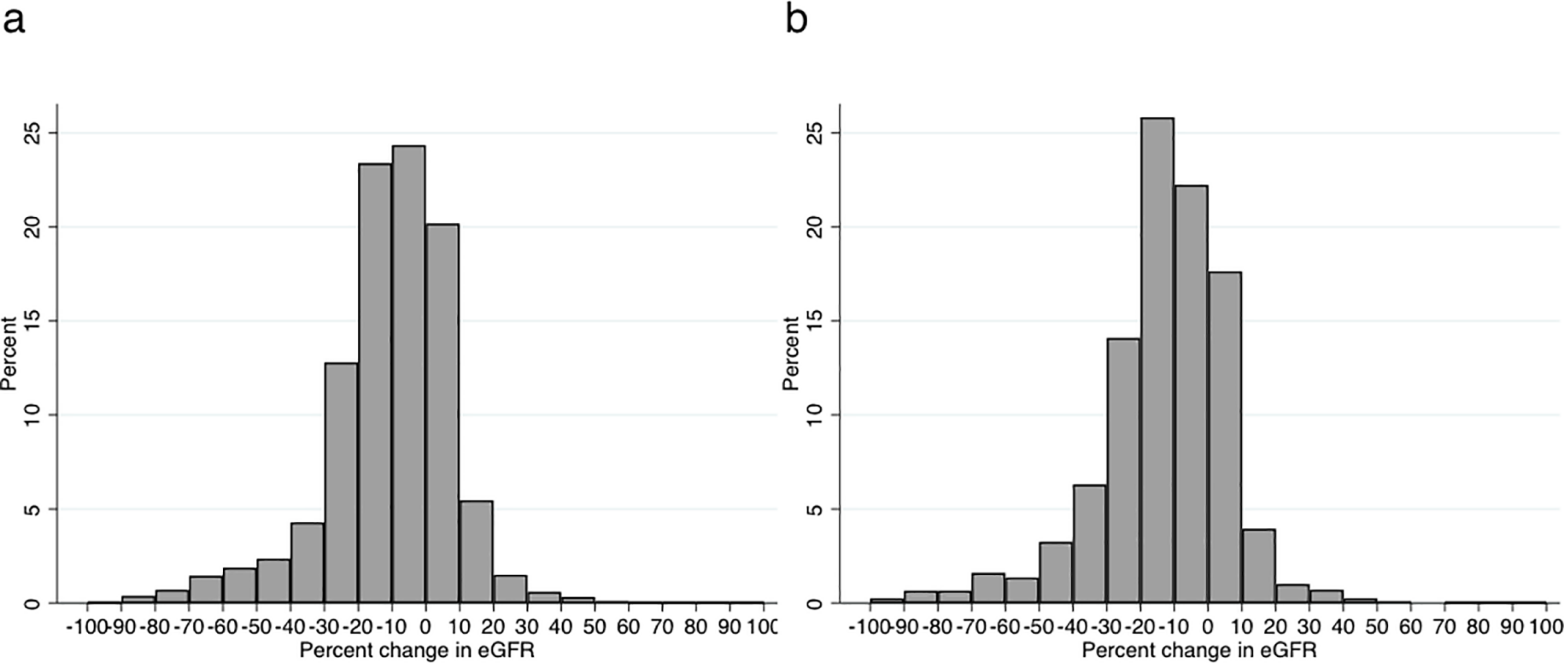
**Distribution of percent changes in eGFR during the (a) 2-year or (b) 3-year baseline period.** Percent change in eGFR was calculated as (last eGFR at baseline period–first available eGFR) / (first available eGFR) * 100.

During the 2- and 3-year baseline period, median changes in eGFR were −11% (5^th^–95^th^ percentile; −48% to +14%) and −13% (−48% to +12%), respectively. The distribution was negatively skewed (Fig 1).

Among the 1868 patients in the 2-year baseline analysis, the prevalence of ≤−20%, ≤−30%, ≤−40%, and ≤−53% changes in eGFR were 23.9% (446 patients), 11.1% (207 patients), 6.8% (127 patients), and 3.7% (70 patients), respectively (Table A in S1 Table). In the 3-year baseline analysis, a slightly larger number of people achieved the surrogate endpoints (S1B Table). Patients with a greater decline in eGFR presented with higher proteinuria and HbA1c, and lower eGFR (Table 2A). There were 133 cases of ESRD (7.1%) during the median follow-up of 6.5 years, and the overall incidence rate of ESRD was 8.6 per 1000 person-years. Among the 133 cases with ESRD, 60.1% (80 cases) and 36.1% (48 cases) reached ≤−30% and ≤−53% changes in eGFR, respectively.

**Table 2:** Baseline characteristics according to percent changes in eGFR during the (a) 2-year or (b) 3-year baseline period.

	Percent changes in eGFR (%)			
	≤ −53	> −53, ≤ −30	> −30, ≤ 0	> 0
**(a) 2-year baseline period (n = 1868)**				
N	70	137	1222	392
ESRD events (n [%])	48 (68.6)	32 (23.3)	47 (3.7)	6 (1.5)
Death (n [%])	4 (5.7)	16 (11.7)	75 (6.1)	34 (8.7)
Age (years; mean [SD])	55 (12.1)	61 (11.8)	61 (11.0)	60 (12.1)
Men (n [%])	42 (60.0)	82 (59.9)	723 (57.0)	223 (56.9)
UACR (mg/g; median [IQR])	2377 (1230, 4019)	236 (25, 1284)	18 (9, 68)	17 (8, 59)
eGFR (mL/min/1.73 m^2^; mean [SD])	58 (26.8)	72 (36.2)	80 (23.1)	71 (22.9)
Systolic BP (mmHg; mean [SD])	140 (22.7)	138 (21.3)	133 (19.5)	132 (19.0)
Diastolic BP (mmHg; mean [SD])	78 (13.4)	78 (11.4)	76 (28.0)	76 (11.2)
History of CVD (n [%])	7 (10.0)	16 (11.7)	131 (10.3)	35 (8.9)
HbA1c (%; mean [SD])	8.6 (2.3)	8.4 (2.2)	8.0 (1.7)	7.8 (1.8)
Follow-up (years; mean [SD])	5.1 (2.4)	5.2 (2.0)	6.6 (3.3)	6.8 (3.6)
**(b) 3-year baseline period (n = 2001)**				
N	79	202	1385	335
ESRD events (n [%])	46 (58.2)	29 (14.4)	33 (2.4)	2 (0.6)
Death (n [%])	10 (12.7)	22 (10.9)	69 (5.0)	27 (8.1)
Age (years; mean [SD])	57 (12.0)	61 (12.1)	61 (10.8)	59 (11.9)
Men (n [%])	52 (65.8)	109 (54.0)	797 (57.6)	201 (60.0)
UACR (mg/g; median [IQR])	1925 (478, 3329)	98 (18, 690)	17 (9, 56)	15.3 (8, 44)
eGFR (mL/min/1.73 m^2^; mean [SD])	58 (26.2)	79 (31.4)	80 (22.8)	72 (22.1)
Systolic BP (mmHg; mean [SD])	139 (21.6)	137 (20.5)	132 (19.1)	131 (17.8)
Diastolic BP (mmHg; mean [SD])	80 (11.3)	75 (11.0)	76 (27.0)	75 (11.4)
History of CVD (n [%])	9 (11.4)	24 (11.9)	124 (9.0)	27 (8.1)
HbA1c (%; mean [SD])	8.4 (2.1)	8.3 (2.0)	7.9 (1.7)	7.5 (1.7)
Follow-up (years; mean [SD])	4.2 (1.8)	4.5 (2.2)	5.7 (3.1)	5.7 (3.4)

ESRD, end-stage renal disease; UACR, urine albumin-to-creatinine ratio; eGFR, estimated glomerular filtration rate; BP, blood pressure; CVD, cardiovascular disease; SD, standard deviation; IQR, interquartile range.

Among the 2001 participants in the 3-year baseline analysis, the prevalence of ≤−20%, ≤−30%, ≤−40%, and ≤−53% changes in eGFR were 28.1% (563 patients), 14.0% (281 patients), 7.7% (155 patients), and 3.9% (79 patients), respectively (Table B in S1 Table). The same trends in baseline characteristics were observed as with the 2-year baseline analysis (Table 2B). There were 110 ESRD cases (5.5%) during the median follow-up of 5.5 years, and the overall incidence rate of ESRD was 6.5 per 1000 person-years. Among the 110 cases with ESRD, 68.1% (75 cases) and 41.8% (46 cases) reached ≤−30% and ≤−53% changes in eGFR, respectively.

### ESRD risk according to percent changes in eGFR

**Fig 2:**
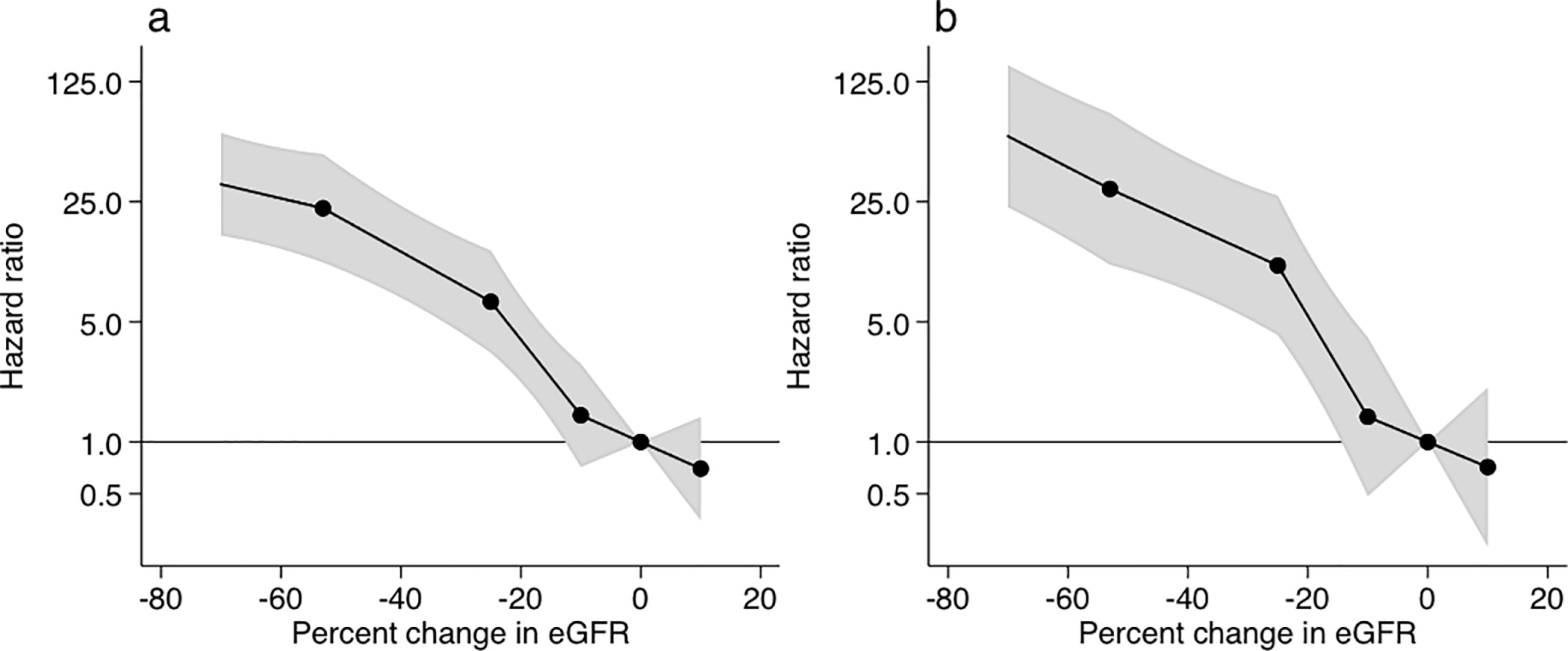
**Adjusted hazard ratios of end-stage renal disease according to percent changes in eGFR during the (a) 2-year or (b) 3-year baseline period.** The model was adjusted for gender, age, systolic blood pressure, baseline eGFR, baseline urinary albumin, and history of cardiovascular disease. Knots were placed at −53%, −25%, −10%, and 10%. A reference point was set at 0% change in eGFR. Value trimmed at less than −70% and >10% change in eGFR.

**Fig 3:**
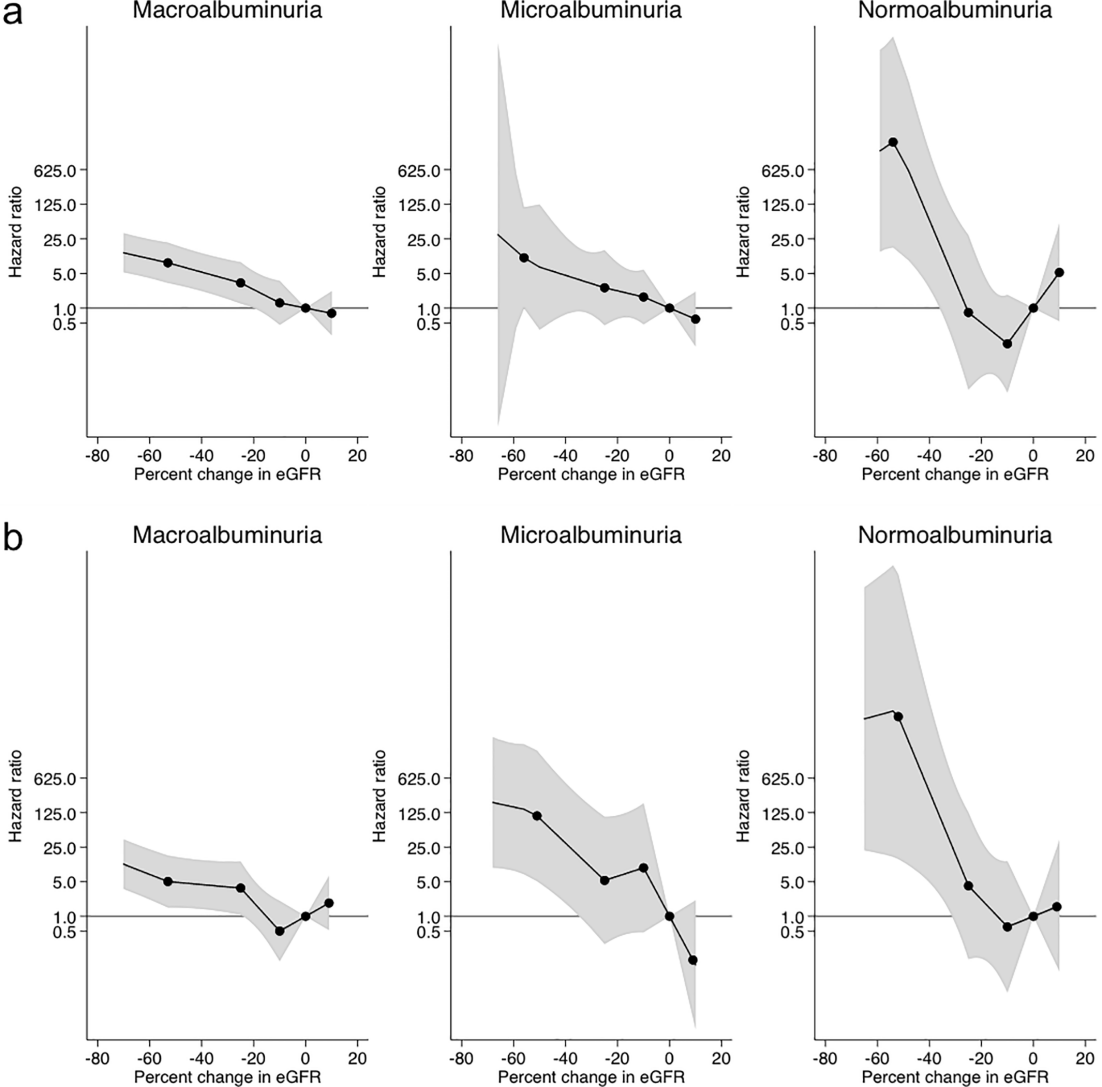
**Adjusted hazard ratios of end-stage renal disease according to percent changes in eGFR during the (a) 2-year or (b) 3-year baseline period in subgroup analysis based on baseline urinary albumin levels.** The model was adjusted with gender, age, systolic blood pressure, baseline eGFR, baseline urinary albumin, and history of cardiovascular disease. Knots were placed at −53%, −25%, −10%, and 10%. A reference point was set at 0% change in eGFR. Value trimmed at less than −70% and >10% change in eGFR.

The risks for ESRD are shown according to the achieved percent changes in eGFR during the 2-year (Fig 2A) or 3-year (Fig 2B) baseline period. In comparison to the no changes in eGFR, adjusted HRs of ESRD were exponentially higher with greater decline in eGFR. Particularly, the −53% change in eGFR over the 2-year baseline period was associated with a 22.9 (11.1–47.3) times higher risk of ESRD when compared with the 0% change in eGFR; the −40%, −30%, and −20% changes in eGFR were associated with 12.8 (6.9–23.7), 8.2 (4.3–15.5), and 3.9 (2.2–7.0) times higher risks of ESRD, respectively (S2 Table). In the 3-year baseline analysis, the corresponding numbers for the −53%, −40%, −30%, and −20% changes in eGFR were 29.7 (10.8–81.9), 18.4 (7.6–44.7), 12.8 (5.2–32.2), and 5.4 (2.3–12.8), respectively (S2 Table). In addition to greater declines in eGFR, the risk of ESRD was significantly elevated with some baseline covariates such as younger age, men, higher proteinuria, systolic BP, and HbA1c, and lower eGFR in multivariable Cox proportional hazard models (S3 Table).

Next, we conducted a subgroup analysis based on baseline UACR category (Fig 3 and S2 Table). In the macroalbuminuria subgroup, as well as in all participants, the decline in eGFR values over the 2- and 3-year periods was significantly associated with subsequent ESRD risk, which grew exponentially with the decline in eGFR when compared with the no change in eGFR. Adjusted HRs for -53%, −40%, −30%, and −20% eGFR changes over the 2-year baseline period were 8.2 (3.2–20.9), 5.3 (2.2–12.8), 3.8 (1.5–9.5), and 2.4 (1.0–5.5), respectively. In the 3-year baseline analysis, the corresponding numbers were 5.1 (1.5–17.0), 4.4 (1.4–13.7), 3.9 (1.2–12.8), and 1.9 (0.7–5.4), respectively. On the other hand, in the microalbuminuria and normoalbuminuria subgroups, a −53% change in eGFR over 2 and 3 years was strongly related to the subsequent risk of ESRD. A considerably lower number of cases with microalbuminuria and normoalbuminuria progressed to ESRD (18 and 5 cases in the 2-year baseline analysis, respectively) when compared to those with macroalbuminuria (87 cases).

**Fig 4:**
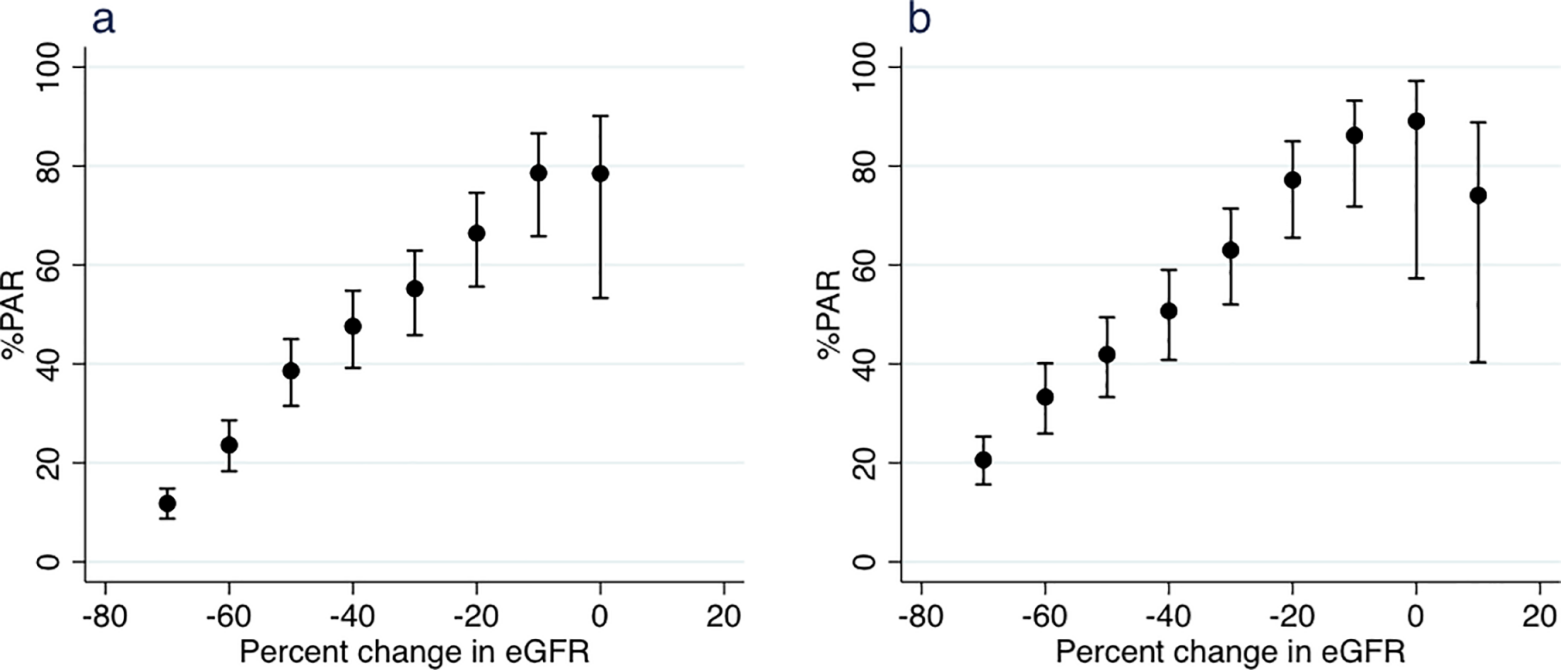
Percent population attributable risk (%PAR) of end-stage renal disease associated with percent changes in eGFR during the (a) 2-year or (b) 3-year baseline period.

The %PAR and PPV of ESRD associated with percent changes in eGFR during the 2- and 3-year baseline periods were calculated (Fig 4 and Table 3). As a result, the cumulative %PAR of ESRD increased remarkably from ≤−70% to ≤−10% change in eGFR, indicating that the smaller the decline in eGFR the higher the prevalence of subsequent ESRD. Specifically, in the 2-year baseline analysis, ≤−20%, ≤−30%, and ≤−40% eGFR changes accounted for 66.4%, 55.2%, and 47.6% of the ESRD events, whereas ≤−53% eGFR change accounted for 33.6% of the ESRD events. In the 3-year baseline analysis, the corresponding numbers were 77.2%, 63.0%, 50.7%, and 39.4%. The PPVs for ESRD in participants with ≤−20%, ≤−30%, ≤−40%, and ≤−53% eGFR changes over the 2-year baseline period were 22.2%, 38.6%, 53.5%, and 68.6%, respectively. Higher PPVs for ESRD were found in participants in the 2-year baseline analysis when compared to those in the 3-year baseline analysis.

**Table 3:** Positive predictive value (PPV) with 95% confidence interval for end-stage renal disease according to percent changes in eGFR during the 2-year and 3-year baseline periods.

	PPV (%) according to percent changes in eGFR			
	≤ −53%	≤ −40%	≤ −30%	≤ −20%
**2-year baseline period**	68.6 (56.4–79.1)	53.5 (44.5–62.4)	38.6 (32.0–45.6)	22.2 (18.4–26.3)
**3-year baseline period**	58.2 (46.6–69.2)	38.7 (31.0–46.9)	26.7 (21.6–32.3)	16.3 (13.4–19.7)

eGFR, estimated glomerular filtration rate.

### All-cause mortality according to percent changes in eGFR

**Fig 5:**
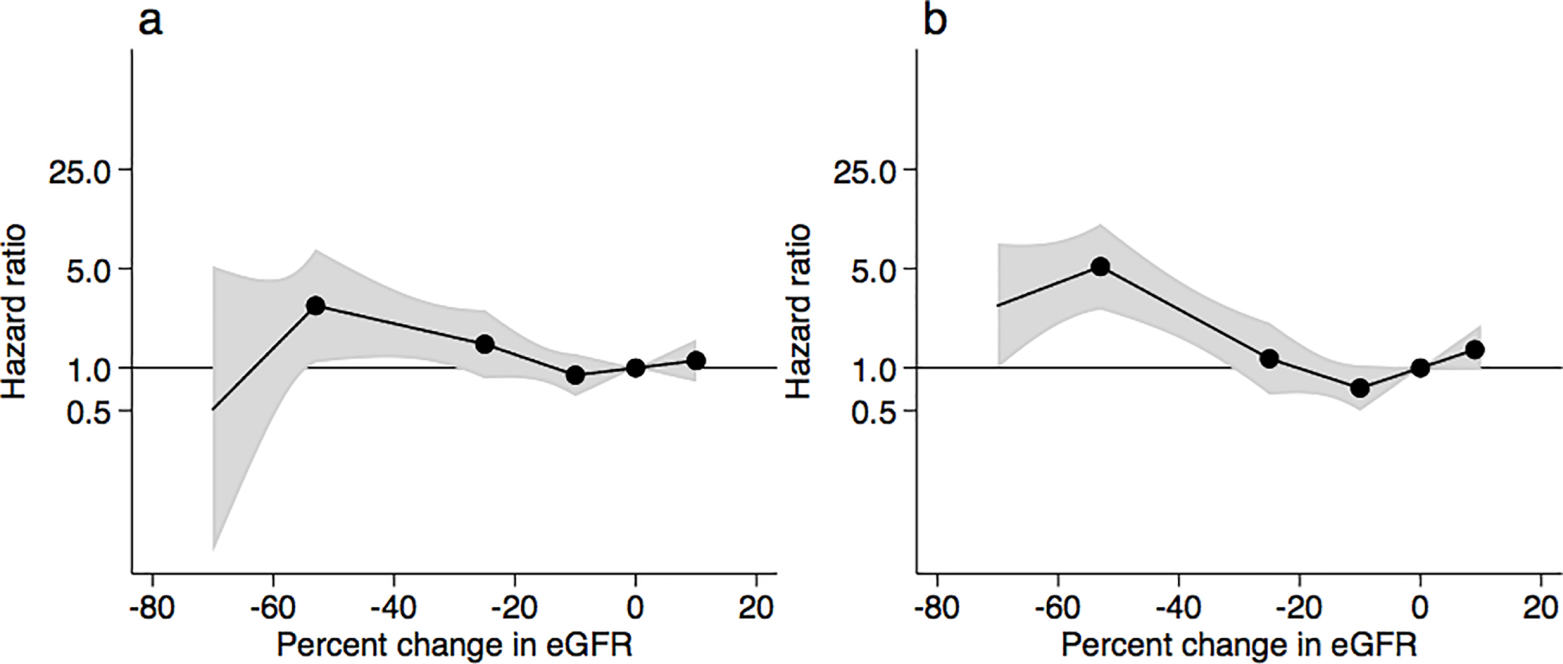
**Adjusted hazard ratios of all-cause mortality according to percent changes in eGFR during the (a) 2-year or (b) 3-year baseline period.** The model was adjusted for gender, age, systolic blood pressure, baseline eGFR, baseline urinary albumin, and history of cardiovascular disease. Knots were placed at −53%, −25%, −10%, and 10%. A reference point was set at 0% change in eGFR. Value trimmed at less than −70% and >10% change in eGFR.

We performed statistical analysis to evaluate the association of percent changes in eGFR with all-cause mortality. In the 2-year baseline analysis, there were 129 cases of all-cause death (6.9%) during the median follow-up of 6.5 years and the overall incidence rate of death was 8.1 per 1000 person-years. Among the 129 cases with all-cause mortality, 15.5% (20 cases) and 3.1% (4 cases) reached ≤−30% and ≤−53% changes in eGFR, respectively. In the 3-year baseline analysis, there were 128 (6.4%) death during the median follow-up of 5.5 years and the overall incidence rate of death was 7.3 per 1000 person-years. The risks for all-cause mortality are shown according to the achieved percent changes in eGFR during the 2-year (Fig 5A) or 3-year (Fig 5B) baseline period. The −53% change in eGFR over the 2-year baseline period was associated with a 2.7 (1.1–6.8) times higher risk of all-cause death when compared with the 0% change in eGFR; the −40% and −30% changes in eGFR were associated with 2.0 (1.2–3.7) and 1.6 (1.0–2.7) times higher risks of all-cause mortality, respectively (S4 Table). In the 3-year baseline analysis, the corresponding numbers for the −53%, −40%, and −30% changes in eGFR were 5.2 (2.6–10.3), 2.6 (1.6–4.1), and 1.5 (0.9–2.5), respectively (S4 Table).

## Discussion

In this study, we examined the possibility of a decrease in eGFR as a surrogate endpoint for ESRD in Japanese type 2 diabetes patients over a baseline period of 2 or 3 years. In 2014, the NKF-FDA committee proposed 30% and 40% declines in eGFR as novel surrogate endpoints for CKD progression in clinical trials, based on the results of some meta-analyses and simulation studies [7, 12–14]. The committee stated that a 30% eGFR decline could be useful under some circumstances; however, a 40% decline in eGFR might be more acceptable across a wider range of baseline GFRs and patterns of treatment effects on GFR [10]. Because 30% and 40% declines in eGFR occur sooner and more frequently than the doubling of sCr, the use of these declines as endpoints in clinical trials under appropriate conditions might reduce follow-up durations, costs, and the number of participants. The present study provided a basis for potential use of the 20% decline in eGFR, in addition to 30% and 40% declines in eGFR, as surrogate endpoints of ESRD among Japanese patients with type 2 diabetes.

The findings of the current study are in accordance with those reported in several previous studies; we have previously published the clinical impact of reduced eGFR on renal events in type 2 diabetic cohort studies [22–25]. The NKF-FDA working group reported the use of eGFR declines as surrogate outcomes in a post-hoc analysis of RCTs and other simulation studies [7, 12–14]. Another research suggested the usefulness of eGFR declines as alternative endpoints in two clinical trials of angiotensin receptor blockers in patients with type 2 diabetes and nephropathy [26]. Matsushita, et al. reported that eGFR reductions over 1 to 2 years were strongly associated with a subsequent risk of ESRD in Japanese patients with CKD, and these associations were consistent across 740 patients with diabetes [17]. These results were suggested based on very well-controlled patients. This study demonstrated the possibility of surrogate outcomes in cohort studies in closer subjects to the real world.

The present study has an advantage in providing analysis stratified by albuminuria at baseline, by conducting a large-scale research focusing on diabetic patients. We observed that eGFR declines were considerably related to ESRD risk in type 2 diabetes patients with macroalbuminuria. This observation is consistent with other studies, which reported that the rapid decline in eGFR could predict the risk of ESRD in type 1 diabetes patients with macroalbuminuria [27–29]. On the other hand, the benefit of using smaller eGFR declines as surrogate endpoints remains unclear among patients with normoalbuminuria and microalbuminuria, partly because of the small number of cases with ESRD for the subgroup analysis. If small-scale and short-term intervention studies are to be carried out for these subgroups, it will be necessary to choose a group with a high risk of event occurrences as reported previously [30, 31].

We estimate that the possibility of surrogate endpoints obtained in the current study can be applied to diabetic patients all over the world, especially to Asian patients who show a high prevalence of albuminuria [32, 33]. The distribution of age and gender in our data was almost same as another nationwide Japanese study including 44255 diabetic patients [34]. Especially, the proportion of people with younger age was not so different from previous reports [35, 36]. Furthermore, the incidence of ESRD is relatively high in Asian people with diabetes [37]. In a cohort study of 64211 diabetic patients including 6901 Asians in California, the incidence rate of ESRD was higher for Asian groups (6.5 per 1000 person-years) compared with whites (3.9 per 1000 person-years) [38]. This rate was almost the same as our results. Also, the incidence rate of ESRD according to the levels of albuminuria were to the same extent as the previous reports [39]. Therefore, the results of the current study might be applicable to trials including Asian people.

Declines in eGFR occurred more commonly and were associated with the risk of mortality, as previously reported [12]. In the 2-year baseline analysis, the -30%, -40%, and -53% changes in eGFR, in compared to no changes in eGFR, correlated to the risk of death, while similar results were obtained in -40% and -53% changes in eGFR from the 3-year baseline analysis. However, there was not significant association with mortality in -20% eGFR changes and we could not indicate the potential use of a lower cut-off value of declines in eGFR to predict the risk of mortality in Japanese patients with type 2 diabetes. Although the risks of all-cause mortality were weaker than those of ESRD, these findings are consistent with other studies including international meta-analysis [12]. In addition, more people achieved mortality outcome in the group of ≤-30% changes in eGFR than the group of ≤-53% changes in eGFR in the 2-year baseline analysis. The main reason for the discrepancy is that there were more participants with high risk factors for death such as older age in the former group, compared with the latter group.

There are several limitations in this study. Firstly, the number of the patients is relatively small, especially in the subgroup analysis. Therefore, the usefulness of surrogate endpoints in patients with normoalbuminuria and microalbuminuria may be unclear due to insufficient detection power. Secondly, although we adjusted for multiple confounding factors, we cannot exclude residual confounding such as body mass index, smoking status, and medications. Especially, in the current study, there is lack of data regarding the medications for blood pressure and glucose control to analyze the treatment effects. In addition, variability in sCr measurements among centers must be considered; moreover, we did not measure sCr regularly. These issues can lead to less accurate calculations of the percent changes in eGFR and reduce the statistical power of the analyses. Thirdly, although this study excluded people who reached ESRD within the 2- or 3-year baseline period from the analysis, we did not distinguish participants who chronically achieved ESRD from those who had acute kidney injury in the follow-up periods, which might result in somewhat imprecise inference about the relationships between chronic changes in eGFR and ESRD. However, the ability to demonstrate the usefulness of surrogate endpoints in a situation close to the actual clinical site indicates its robustness.

In conclusion, our study revealed that eGFR declines were strongly associated with subsequent risk of ESRD in Japanese type 2 diabetic patients. In addition to 30% and 40% declines in eGFR, a 20% decline in eGFR over 2 years could be considered as a candidate surrogate endpoint of ESRD in DKD. These results were confirmed mainly in patients with macroalbuminuria. In future, simulation studies, as described in the accompanying articles [13,14], may be helpful to evaluate the validity of these surrogate endpoints.

## Supporting information

S1 Fig Flowchart of constructing our cohort. Abbreviations: UACR, urine albumin-to-creatinine ratio; BP, blood pressure; ESRD, end-stage renal disease; sCr, serum creatinine.
(TIFF)

S1 Table Baseline characteristics according to percent changes in eGFR during the (a) 2-year or (b) 3-year baseline period.
(PDF)

S2 Table Adjusted hazard ratios of end-stage renal disease according to percent changes in eGFR during the 2-year or 3-year baseline period referred to no change in eGFR. (PDF)

S3 Table Multivariate Cox proportional hazards models of risk factors for end-stage renal disease in the 2-year or 3-year baseline analysis. (PDF)

S4 Table Adjusted hazard ratios of all-cause mortality according to percent changes in eGFR during the 2-year or 3-year baseline period referred to no change in eGFR. (PDF)
