# Supplementary figures and images for: Estimated glomerular filtration rate decline and risk of end-stage renal disease in type 2 diabetes
Source: PLoS One. 2018 Aug 2;13(8):e0201535. doi: 10.1371/journal.pone.0201535 (PMC6072050; doi:10.1371/journal.pone.0201535)

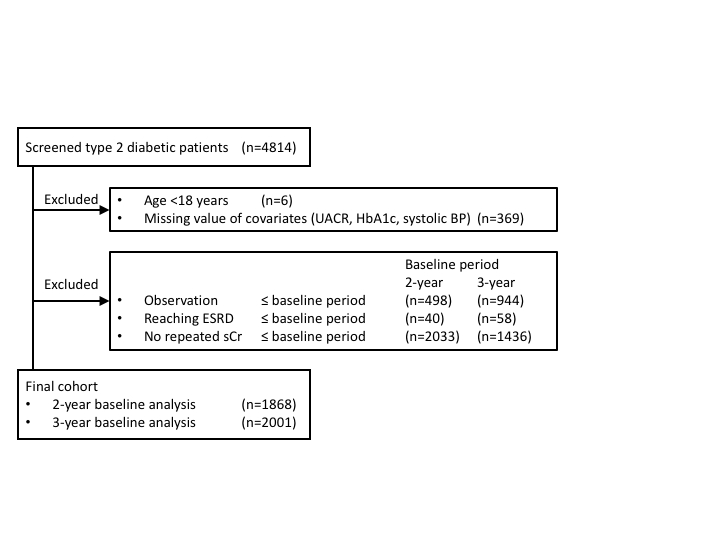

Supplement: S1 Fig — Abbreviations: UACR, urine albumin-to-creatinine ratio; BP, blood pressure; ESRD, end-stage renal disease; sCr, serum creatinine. (TIFF) [file pone.0201535.s001.tiff]
