# Supplementary material for: Estimated glomerular filtration rate decline and risk of end-stage renal disease in type 2 diabetes
Source: PLoS One. 2018 Aug 2;13(8):e0201535. doi: 10.1371/journal.pone.0201535 (PMC6072050; doi:10.1371/journal.pone.0201535)
Supplement: S1 Table — Baseline characteristics according to percent changes in eGFR during the (a) 2-year or (b) 3-year baseline period. (PDF) [file pone.0201535.s002.pdf]

S1 Table. Baseline characteristics according to percent changes in eGFR during the (a) 2-year or (b) 3-year baseline period.

(a) 2-year baseline period (n=1868)

	Percent changes in eGFR			
	**≤ −53%**	**≤ −40%**	**≤ −30%**	**≤ −20%**
**N**	70	127	207	446
**ESRD events (n [%])**	48 (68.6)	68 (53.5)	80 (38.7)	99 (22.2)
**Incidence rate of ESRD**	139.1	99.4	64.5	32.0
**(per 1000 person-years [95%CI])**	(104.8–184.6)	(78.4–126.1)	(51.8–80.3)	(26.2–38.9)
**Death (n [%])**	4 (5.7)	10 (7.9)	20 (9.7)	36 (8.1)
**Incidence rate of death**	8.1	11.2	13.5	10.7
**(per 1000 person-years [95%CI])**	(6.9–9.7)	(6.0–20.9)	(8.7–20.9)	(7.7–14.9)
**Age (years; mean [SD])**	55 (12.1)	58 (11.9)	59 (12.1)	60 (11.9)
**Men (n [%])**	42 (60.0)	80 (63.0)	124 (59.9)	246 (55.2)
**UACR (mg/g; median [IQR])**	2378<br>(1230, 4019)	1731<br>(478, 3684)	825<br>(61, 2621)	90<br>(15, 1152)
**eGFR (ml/min/1.73 m^2; mean [SD])**	58 (26.8)	61 (33.6)	68 (33.9)	76 (31.4)
**Systolic BP (mmHg; mean [SD])**	140 (22.7)	141 (21.7)	139 (21.7)	136 (21.0)
**Diastolic BP (mmHg; mean [SD])**	78 (13.4)	79 (12.9)	78.1 (12.1)	77 (12)
**History of CVD (n [%])**	7 (8.6)	14 (11.0)	22 (11.1)	56 (12.6)
**HbA1c (%; mean [SD])**	8.6 (2.3)	8.5 (2.2)	8.5 (2.3)	8.5 (2.0)
**Death (n [%])**	4 (5.7)	10 (7.9)	20 (9.7)	36 (8.1)
**Follow-up (years; mean [SD])**	5.1 (2.4)	5.0 (2.2)	5.1 (2.2)	5.5 (2.6)

(b) 3-year baseline period (n=2001)

	Percent changes in eGFR			
	**≤ −53%**	**≤ −40%**	**≤ −30%**	**≤ −20%**
**N**	79	155	281	563
**ESRD events (n [%])**	46 (58.2)	60 (38.7)	75 (26.7)	92 (16.3)
**Incidence rate of ESRD**	100.7	61.5	39.1	22.1
**(per 1000 person-years [95%CI])**	(75.4–134.4)	(47.7–79.2)	(31.2–49.0)	(18.0–27.1)
**Death (n [%])**	10 (12.7)	23 (14.8)	32 (11.4)	51 (9.1)
**Incidence rate of death**	25.6	24.7	16.7	12.1
**(per 1000 person-years [95%CI])**	(13.3–49.1)	(16.2–37.5)	(11.7–23.7)	(9.2–8.7)

**Age (years; mean [SD])**	57 (12.0)	58 (12.7)	60 (12.2)	60 (11.5)
**Men (n [%])**	52 (65.8)	90 (58.1)	161 (57.3)	315 (56.0)
**UACR (mg/g; median [IQR])**	1925 (478, 3329)	758 (63, 2378)	226 (25, 1446)	55 (14, 598)
**eGFR (mL/min/1.73 m^2; mean [SD])**	58 (26.2)	69 (33.5)	73 (31.4)	78 (29.7)
**Systolic BP (mmHg; mean [SD])**	139 (21.6)	139 (20.7)	137 (20.8)	135 (20.6)
**Diastolic BP (mmHg; mean [SD])**	80 (11.3)	78 (11.6)	77 (11.2)	76 (11.5)
**History of CVD (n [%])**	9 (11.4)	18 (11.6)	33 (11.7)	70 (12.4)
**HbA1c (%; mean [SD])**	8.4 (2.1)	8.5 (2.1)	8.4 (2.0)	8.4 (2.0)
**Follow-up (years; mean [SD])**	4.2 (1.8)	4.2 (1.7)	4.4 (2.1)	4.7 (2.5)

ESRD, end-stage renal disease; CI, Confidence interval; UACR, urine albumin-to-creatinine ratio; eGFR, estimated glomerular filtration rate; BP, blood pressure; CVD, cardiovascular disease; SD, standard deviation; IQR, interquartile range.
