# Supplementary material for: Estimated glomerular filtration rate decline and risk of end-stage renal disease in type 2 diabetes
Source: PLoS One. 2018 Aug 2;13(8):e0201535. doi: 10.1371/journal.pone.0201535 (PMC6072050; doi:10.1371/journal.pone.0201535)
Supplement: S2 Table — (PDF) [file pone.0201535.s003.pdf]

S2 Table. Adjusted hazard ratios of end-stage renal disease according to percent changes in eGFR during the 2-year or 3-year baseline period referred to no change in eGFR.

	Percent changes in eGFR				
	**−53%**	**−40%**	**−30%**	**−20%**	**0% Reference**
**2-year baseline period**					
**All participants**	22.9 (11.1–47.3)	12.8 (6.9–23.7)	8.2 (4.3–15.5)	3.9 (2.2–7.0)	1
**Macroalbuminuria**	8.2 (3.2–20.9)	5.3 (2.2–12.8)	3.8 (1.5–9.5)	2.4 (1.0–5.5)	1
**Microalbuminuria**	10.4 (1.0–108.0)	4.6 (0.8–27.9)	3.1 (0.7–13.4)	2.2 (0.6–8.2)	1
**Normoalbuminuria**	2245.5 (16.8–299867.1)	78.9 (3.0–2060.4)	3.4 (0.2–75.1)	0.5 (0.04–6.2)	1
**3-year baseline period**					
**All participants**	29.7 (10.8–81.9)	18.4 (7.6–44.7)	12.8 (5.2–32.2)	5.4 (2.3–12.8)	1
**Macroalbuminuria**	5.1 (1.5–17.0)	4.4 (1.4–13.7)	3.9 (1.2–12.8)	1.9 (0.7–5.4)	1
**Microalbuminuria**	107.7 (5.1–2254.4)	30.2 (1.8–498.1)	9.5 (0.5–164.0)	6.5 (0.4–110.1)	1
**Normoalbuminuria**	10956.6 (14.5–8299862.5)	329.9 (4.8–22462.8)	17.8 (0.7–450.2)	2.2 (0.1–34.1)	1

eGFR, estimated glomerular
