# Supplementary material for: Estimated glomerular filtration rate decline and risk of end-stage renal disease in type 2 diabetes
Source: PLoS One. 2018 Aug 2;13(8):e0201535. doi: 10.1371/journal.pone.0201535 (PMC6072050; doi:10.1371/journal.pone.0201535)
Supplement: S3 Table — (PDF) [file pone.0201535.s004.pdf]

S3 Table. Multivariate Cox proportional hazards models of risk factors for end-stage renal disease in the 2-year or 3-year baseline analysis.

	2-year baseline analysis		3-year baseline analysis	
	Hazard ratio	P value	Hazard ratio	P value
**Percent changes in eGFR (%)**				
**> 0**	1		1	
**> −30, ≤ 0**	2.67 (1.13–6.29)	0.03	4.49 (1.07–18.78)	0.04
**> −53, ≤ −30**	13.73 (5.35–35.22)	<0.01	18.56 (4.24–81.15)	<0.01
**≤ −53**	28.27 (10.79–74.06)	<0.01	47.38 (10.43–215.22)	<0.01
**Baseline characteristics**				
**Age (+10 years)**	0.63 (0.52–0.77)	<0.01	0.69 (0.56–0.86)	<0.01
**Men (vs. Women)**	1.55 (1.04–2.30)	0.03	1.41 (0.90–2.20)	0.14
**UACR (+10 mg/g)**	1.00 (1.00–1.00)	<0.01	1.00 (1.00–1.00)	<0.01
**eGFR (-10 mL/min/1.73 m^2)**	1.56 (1.41–1.73)	<0.01	1.43 (1.29–1.59)	<0.01
**Systolic BP (+10 mmHg)**	1.18 (1.08–1.28)	<0.01	1.19 (1.07–1.31)	<0.01
**HbA1c (+1.0 %)**	1.26 (1.14–1.39)	<0.01	1.26 (1.13–1.40)	<0.01

Abbreviations: eGFR, estimated glomerular filtration rate; UACR, urine albumin-to-creatinine ratio; BP, blood pressure.
