# Supplementary material for: Estimated glomerular filtration rate decline and risk of end-stage renal disease in type 2 diabetes
Source: PLoS One. 2018 Aug 2;13(8):e0201535. doi: 10.1371/journal.pone.0201535 (PMC6072050; doi:10.1371/journal.pone.0201535)
Supplement: S4 Table — (PDF) [file pone.0201535.s005.pdf]

S4 Table. Adjusted hazard ratios of all-cause mortality according to percent changes in eGFR during the 2-year or 3-year baseline period referred to no change in eGFR.

	Percent changes in eGFR				
	**−53%**	**−40%**	**−30%**	**−20%**	**0% (Reference)**
**2-year baseline period**	2.7 (1.1–6.8)	2.0 (1.2–3.6)	1.6 (1.0–2.7)	1.2 (0.9–1.8)	1
**3-year baseline period**	5.2 (2.6–10.3)	2.6 (1.6–4.1)	1.5 (0.9–2.5)	1.0 (0.7–1.5)	1

eGFR, estimated glomerular filtration rate
